# Access to Information Cited in National Organization for Rare Disorders Reports

**DOI:** 10.1001/jamanetworkopen.2025.11758

**Published:** 2025-05-21

**Authors:** Mengyuan Fu, Kexin Ling, Xinyi Zhou, Sneha Dave, Can Li, Luwen Shi, Xiaodong Guan, Joseph S. Ross

**Affiliations:** 1 Department of Internal Medicine, Yale School of Medicine, New Haven, Connecticut; 2 International Research Center for Medicinal Administration, Peking University, Beijing, China; 3 Department of Pharmacy Administration and Clinical Pharmacy, School of Pharmaceutical Sciences, Peking University, Beijing, China; 4 Generation Patient, Indianapolis, Indiana; 5 Center for Outcomes Research and Evaluation, Yale-New Haven Health System, New Haven, Connecticut; 6 Department of Health Policy and Management, Yale School of Public Health, New Haven, Connecticut

## Abstract

This cross-sectional study describes the availability and access cost to the public of research findings and other clinical insights relevant to rare diseases.

## Introduction

Over the past 20 years, substantial steps have been taken to ensure public access to research results, including policies established by the US Office of Science and Technology Policy^1,2^ and the National Institutes of Health.^3,4^ Patients with rare diseases and their caregivers play outsized roles in their medical care due to limited treatment options and specialists. Patient organizations, such as the National Organization for Rare Disorders (NORD), are essential to facilitating access to high-quality information for families.^5^ While NORD’s disease-specific reports are publicly available, they provide only brief overviews; patients may seek the detailed information and research findings cited within the reports to inform clinical decision-making. Accordingly, this study examined the public availability and cost of accessing these cited information sources.

## Methods

In accordance with the Common Rule, this cross-sectional study was exempt from ethics review and informed consent because it used public, nonidentifiable data and was not human participant research. We followed the STROBE reporting guideline.

We identified all NORD rare-disease reports available as of February 2024 and randomly selected 20% for analysis. Information sources cited in these reports were classified into 5 types: research articles, case reports, reviews (all publications in scholarly journals), textbooks, and other internet resources. Next, study team members (M.F., X.Z., K.L., and C.L.) searched (using Google) for these citations and categorized each according to online availability: open access (free), accessible at cost, or unavailable (eMethods in Supplement 1). For citations with associated access costs, we recorded the lowest listed price before taxes. A 10% cross-sample validation was conducted between each pair of researchers to check for consistency. All data were collected between April and May 2024.

We used descriptive statistics to calculate the proportion of citations available (overall and by publication year and information source type) and median access costs (overall and by information source type). We used χ^2^ tests to evaluate the differences of open access rate by year and source type. Two-sided *P* < .05 was considered statistically significant. Analyses were performed using Stata BE, version 16.0 (StataCorp).

## Results

Among 1333 NORD rare-disease reports available by February 2024, 267 (20.0%) were randomly selected. These 267 reports included 4445 citations to 3506 unique information sources. The median (IQR) number of citations per report was 15 (11-20), including 9 (5-13) research articles, 0 (0-1) case reports, 0 (0-0) review articles, 1 (0-3) textbook, and 2 (1-4) internet resources.

Among 3506 unique information sources cited, 1750 (49.9%) were open access, 1381 (39.4%) were accessible at cost, and 375 (10.7%) were unavailable online (Table). Among citations only accessible at cost, the median (IQR) cost was $32.00 ($15.00-$40.75), whereas the median (IQR) total cost per report for all citations only accessible at cost was $167.70 ($82.00-$258.70). Among unique information sources published in scholarly journals, the proportion available as open access increased from 30.0% (258 of 860) for those published before 2001 to 79.8% (95 of 119) for those published in 2021 and afterward (Figure).

**Table.** Public Availability of Information Sources Cited in 267 NORD Reports

Characteristics	Information sources cited, No. (%)						*P* value
	Overall	Scholarly publications			Textbook	Internet resources	
		Research article	Case report	Review			
Public availability							
Open access	1750 (49.9)	1409 (52.0)	113 (41.7)	115 (46.2)	6 (5.0)	107 (67.1)	<.001
Accessible at cost^a^	1381 (39.4)	1074 (39.7)	92 (33.9)	113 (45.4)	102 (85.7)	1 (0.6)	<.001
Pay-per-view access	NA	22 (0.8)	3 (1.1)	1 (0.4)	NA	NA	NA
Limited period of online access	NA	635 (23.4)	53 (19.6)	49 (19.7)	NA	NA	NA
Unlimited online-only access	NA	16 (0.6)	3 (1.1)	0 (0.0)	NA	NA	NA
PDF download access	NA	319 (11.8)	27 (10.0)	54 (21.7)	NA	NA	NA
Specific type not mentioned	NA	81 (3.0)	6 (2.2)	9 (3.6)	NA	NA	NA
Unavailable online	375 (10.7)	225 (8.3)	66 (24.4)	21 (8.4)	11 (9.3)	52 (32.3)	<.001
Total	3506	2707	271	249	119	160	NA
Cost of information sources only accessible at cost, median (IQR), $^b^	32.00 (15.00-40.75)	31.75 (15.00-39.95)	31.50 (13.50-40.75)	35.95 (27.95-40.75)	30.98 (9.03-77.33)	23.0 (NR)	NA

Abbreviations: NA, not applicable; NORD, National Organization for Rare Disorders; NR, not reported.

^a^ Each scholarly publications was assigned to 1 independent subcategory according to which access was at the lowest cost.

^b^ Among citations only accessible at cost, the median (IQR) costs were calculated. Costs were converted into US dollars uniformly on the basis of the exchange rate on May 24, 2024. The IQR was unavailable for internet resources.

**Figure.**
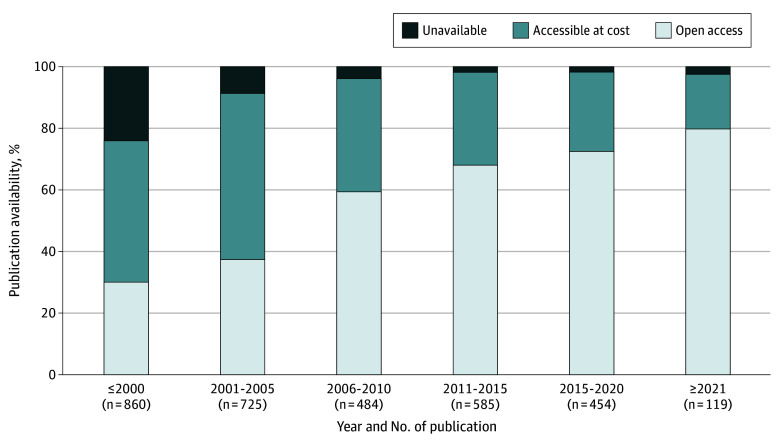
Public Availability of Scholarly Publications Cited in 267 National Organization for Rare Disorders Reports by Year of Publication Scholarly publications include research articles, case reports, and reviews.

## Discussion

This cross-sectional study found that half (50.1%) of information sources cited in NORD rare-disease reports were not publicly available. However, access to scholarly journal publications has significantly improved over time. While NORD should be applauded for making its rare-disease reports publicly available, additional efforts are needed to provide public access to research findings and other information cited in the reports; similar challenges exist for other organizations, such as Orphanet.

Study limitations include that some citations may have been categorized as open access or accessible at lower costs if other search engines were used. Moreover, we did not determine funders for cited studies and thus could not assess adherence to these funders’ open access policies. Nevertheless, these policies are crucial for advancing health equity and expanding access to key information sources that patients may rely on to inform their care.

## Notes

Supplement 1.
eMethods.

Supplement 2.
Data Sharing Statement

## References

[1] Office of Science and Technology Policy. Ensuring free, immediate, and equitable access to federally funded research. Accessed May 1, 2024. https://www.whitehouse.gov/wp-content/uploads/2022/09/M-22-18.pdf

[2] Office of Science and Technology Policy. Increasing access to the results of federally funded scientific research. Accessed May 1, 2024. https://obamawhitehouse.archives.gov/sites/default/files/microsites/ostp/ostp_public_access_memo_2013.pdf

[3] National Institutes of Health. NIH Public access policy. Accessed May 1, 2024. https://sharing.nih.gov/public-access-policy/public-access-policy-overview#public-access-policy-details

[4] National Institutes of Health. 2024 NIH public access policy. Accessed April 8, 2025. https://grants.nih.gov/grants/guide/notice-files/NOT-OD-25-047.html

[5] National Organization for Rare Disorders. Rare disease database. Accessed March 1, 2024. https://rarediseases.org/rare-diseases/

