# Supplementary material for: Access to Information Cited in National Organization for Rare Disorders Reports
Source: JAMA Netw Open. 2025 May 21;8(5):e2511758. doi: 10.1001/jamanetworkopen.2025.11758 (PMC12096240; doi:10.1001/jamanetworkopen.2025.11758)
Supplement: Supplement 1. — eMethods. [file jamanetwopen-e2511758-s001.pdf]

Supplemental Online Content

Fu M, Ling K, Zhou X, et al. Access to information cited in National Organization for Rare Disorders reports. *JAMA Netw Open*. 2025;8(5):e2511758.
doi:10.1001/jamanetworkopen.2025.11758

eMethods.

This supplemental material has been provided by the authors to give readers additional information about their work.

© 2025 Fu M et al. *JAMA Network Open*

eMethods

Study sample

As of the end of Feb 2024, NORD had posted 1,333 unique rare disease reports.[11] Diseases are listed by initial character, A-Z, and in alphabetical order within each initial character, with 15 reports posted per web page (except for the last page). We randomly selected 20% (267/1,333) of the NORD rare disease reports for inclusion, using the page number and report number (e.g. page1-report 14).

Data collection

For each NORD rare disease report, we collected and reviewed all referenced citations, which were classified into 5 source types, including research articles, case reports, review articles, textbooks, and other internet sources in the report. Four members of the study team (M.F., X.Z., K.L. and C.L.) independently extracted, searched, and categorized all citations from 3 sample reports. Discrepancies were reviewed and discussed among the group to reach consensus. Subsequently, these 4 members conducted the main search, categorized citations into different accessibility categories and recorded the lowest listed cost before taxes for those determined to be accessible at cost. A 10% cross-sample validation was conducted between each pair of researchers to check consistency. The consistency rate achieved was 100%. All data on accessibility and cost were collected between April and May, 2024.

Determination of accessibility

a. Research articles published in medical journals

All research articles published in medical journals will be categorized into three accessibility subgroups as follows:

- **a1, Open access:** free reading and downloading PDF to the public, with or without free subscription to the journal;
- **a2, Accessible at cost:** require purchasing reading and/or downloading PDF access to the public;

© 2025 Fu M et al. *JAMA Network Open*

    - a2-1, Pay-per-view access
    - a2-2, Limited period of online access (ie, 24-Hour, 48-Hour, 3-day, 7-day)
    - a2-3, Unlimited online-only access
    - a2-4, PDF download access
    - a2-5, Not mentioned specific type of access on the purchasing page, or only available via institutional subscribe
- **a3, Unavailable:** no full article available online (article page cannot be found, or full article link cannot be found online, or full article not available in English).

b. Case reports published in medical journals

All case reports published in medical journals will be categorized into three accessibility subgroups as follows:

- **b1, Open access:** free reading and downloading PDF to the public, with or without free subscription to the journal;
- **b2, Accessible at cost:** require purchasing reading and/or downloading PDF access to the public;
    - b2-1, Pay-per-view access
    - b2-2, Limited period of online access (ie, 24-Hour, 48-Hour, 3-day, 7-day)
    - b2-3, Unlimited online-only access
    - b2-4, PDF download access
    - b2-5, Not mentioned specific type of access on the purchasing page
- **b3, Unavailable:** no full article available online.

c. Review articles published in medical journals

All review articles published in medical journals will be categorized into three accessibility subgroups as follows:

- **c1, Open access:** free reading and downloading PDF to the public, with or without free subscription to the journal;
- **c2, Accessible at cost:** require purchasing reading and/or downloading PDF access to the public;
    - c2-1, Pay-per-view access
    - c2-2, Limited period of online access (ie, 24-Hour, 48-Hour, 3-day, 7-day)
    - c2-3, Unlimited online-only access
    - c2-4, PDF download access
    - c2-5, Not mentioned specific type of access on the purchasing page
- **c3, Unavailable:** no full article available online.

© 2025 Fu M et al. *JAMA Network Open*

d. Textbooks

Each textbook will be categorized into three accessibility subgroups as follows:

- **d1, Open access:** book can be read online without payment;
- **d2, Accessible at cost:** require purchasing reading to the public, whether it is eBook or printed, new or used;
- **d3, Unavailable:** book not available online, whether it is eBook or printed, new or used.

e. Other internet resources

Other internet resources will be categorized into three accessibility subgroups as follows:

- **e1, Open access**
  - **valid link:** cited link to the report or website is available, or the link directs users to an updated version of the report/website;
  - **invalid link:** cited link to the report or website is invalid (i.e., 404 error) and does not direct users to an updated version of the report/website, but an updated version of the report/website can be found using Google search;
- **e2, Accessible at cost:** require purchasing reading to the public;
- **e3, Unavailable:** cited link to the report or website is invalid (i.e., 404 error) and no updated version of the report/website can be found using Google search.
