# Supplementary material for: Access to Information Cited in National Organization for Rare Disorders Reports
Source: JAMA Netw Open. 2025 May 21;8(5):e2511758. doi: 10.1001/jamanetworkopen.2025.11758 (PMC12096240; doi:10.1001/jamanetworkopen.2025.11758)
Supplement: Supplement 2. — Data Sharing Statement [file jamanetwopen-e2511758-s002.pdf]

Data Sharing Statement

Fu. Access to Information Cited in National Organization for Rare Disorders Reports. *JAMA Netw Open*. Published May 21, 2025. doi:10.1001/jamanetworkopen.2025.11758

Data
Data available: No

Additional Information
Explanation for why data not available: Relevant data are available on reasonable request from the corresponding author (joseph.ross@yale.edu).
